# Supplementary material for: Radiomics and Artificial Intelligence in Uterine Sarcomas: A Systematic Review
Source: J Pers Med. 2021 Nov 11;11(11):1179. doi: 10.3390/jpm11111179 (PMC8624692; doi:10.3390/jpm11111179)
Supplement: Supplementary file 1 [file jpm-11-01179-s001.zip › jpm-1368972-supplementary.pdf]

**Supplementary Table S1.** Boolean combinations of the key terms used	
**Database**	**Free-vocabulary and/or Medical Subject Headings (MeSH) terms**
Pubmed<br>And Cochrane<br>Library	((uterine neoplasms[Title/Abstract] OR uterine sarcomas[Title/Abstract] OR uterine fibroids[Title/Abstract] OR endometrial cancer[Title/Abstract]) OR ("Uterine Neoplasms"[Mesh])) AND ((radiomics[Title/Abstract]) OR (("Artificial Intelligence"[Majr]) OR (robotics[Title/Abstract] OR AI[Title/Abstract] OR expert system[Title/Abstract] OR expert systems[Title/Abstract] OR intelligent retrieval[Title/Abstract] OR knowledge engineering[Title/Abstract] OR machine learning[Title/Abstract] OR natural language processing[Title/Abstract]))) Filters: Female
Scopus	((TITLE-ABS-KEY (image AND interpretation OR "image processing")) AND (TITLE-ABS-KEY ("uterine neoplasms"))) OR (TITLE-ABS-KEY ("medical image analysis")) OR (TITLE-ABS-KEY (radiomics)) AND ((TITLE-ABS-KEY ("uterine neoplasms")) OR (TITLE-ABS-KEY ("endometrial cancer" OR "cervical cancer" OR<br>"uterine sarcomas" OR "uterine fibromas"))) AND (LIMIT-TO (EXACTKEYWORD, "Image Processing, Computer-Assisted"))
